# Sleep-dependent memory consolidation in breast cancer: Use of a virtual reality prospective memory task

**DOI:** 10.3389/fnins.2022.908268

**Published:** 2022-09-07

**Authors:** Mylène Duivon, Joy Perrier, Carine Segura-Djezzar, Florence Joly, Stéphane Rehel, Christian Berthomier, Jean-Michel Grellard, Bénédicte Clarisse, Julien Geffrelot, George Emile, Christelle Lévy, Fausto Viader, Francis Eustache, Béatrice Desgranges, Géraldine Rauchs, Bénédicte Giffard

**Affiliations:** ^1^Neuropsychologie et Imagerie de la Mémoire Humaine, U1077, Normandie University, UNICAEN, PSL Université, EPHE, INSERM, CHU de Caen, GIP Cyceron, Caen, France; ^2^Department of Clinical Research Unit and Medical Oncology, Caen, France; ^3^Institut Normand du Sein, Centre François Baclesse, Caen, France; ^4^U1086 ANTICIPE, INSERM, Normandie University, UNICAEN, Caen, France; ^5^Cancer and Cognition Platform, Ligue Nationale Contre le Cancer, Caen, France; ^6^Physip, Paris, France

**Keywords:** breast cancer, cognition, sleep, polysomnography, prospective memory, memory consolidation, virtual reality

## Abstract

**Background:**

Previous studies have revealed both sleep alterations and prospective memory (PM) impairments in breast cancer (BC) patients. PM refers to memory of intended actions and is crucial for daily living tasks and treatment compliance. As sleep is known to favor memory consolidation, one may expect that changes in sleep quality related to BC would have an impact on PM performance. This study aimed at assessing sleep-dependent consolidation of intentions using an ecological, virtual reality-based PM task in BC patients not treated with chemotherapy.

**Materials and methods:**

Thirty-seven early stages BC patients and 21 healthy controls (HC) participated in this study. PM was assessed using a virtual reality task, during which participants learnt a list of intentions and recalled them after a retention interval filled with a day awake or a night of sleep monitored by polysomnography. Sleep spindles and slow waves, brain oscillations involved in sleep-dependent memory consolidation, were quantified automatically using the Aseega software (Physip). Subjective sleep disturbances and markers of quality of life (psychological distress, fatigue, and well-being) were assessed by questionnaires.

**Results:**

Greater PM performance was observed after sleep than after an equivalent period of daytime wakefulness for both groups (HC and BC). PM performance after sleep did not differ significantly between groups. Yet, BC patients reported greater sleep disturbances than HC which were related with poorer intentions retrieval, greater psychological distress, fatigue and poorer well-being. The frequency of spindles was higher and the amplitude of slow waves lower in BC patients compared to HC. However, no significant association was observed between polysomnography parameters and PM scores in the whole sample of participants.

**Conclusion:**

Although subtle changes in brain oscillations involved in sleep-dependent memory consolidation were observed, these changes did not significantly impair overnight PM consolidation in BC patients. Nevertheless, poorer PM performance was associated with greater sleep complaints which in turn were related to poorer quality of life. Overall, these data suggest that sleep-dependent PM consolidation mechanisms are not altered in early stages BC patients not treated with chemotherapy. Further investigations are needed to understand the association between markers of quality of life and sleep-dependent memory consolidation.

## Introduction

Sleep disturbances and memory impairments are one of the most frequent complaints from breast cancer (BC) patients (Lange et al., 2019; Leysen et al., 2019; Perrier et al., 2021). Sleep complaints are related to greater fatigue and poorer quality of life in cancer survivors (Nishiura et al., 2013; Xu et al., 2018). Previous studies, although scarce and conducted on small samples, suggested that both cancer and chemotherapy treatment might lead to subtle changes in sleep architecture measured by polysomnography (PSG; Roscoe et al., 2011; Tag Eldin et al., 2019). The studies reporting significant changes revealed a decrease in total sleep time with shorter time spent in REM (Rapid-Eye Movement) sleep and longer time spent in lighter sleep stages, i.e., N1 and N2 (Parker et al., 2008; Tag Eldin et al., 2019). A recent study has shown changes in cortical activity during sleep in BC patients not treated with chemotherapy, with notably lower delta power during non-REM sleep in patients compared to healthy controls (HC; Perrier et al., 2022). Delta power is the EEG activity related to slow waves which are, together with sleep spindles, one of the electrophysiological signatures of sleep-dependent memory consolidation (Holz et al., 2012; Klinzing et al., 2019).

According to the “Hippocampo-Neocortical Dialog” hypothesis, both slow waves and spindles are involved in the reorganization of newly acquired information into long-term memory stores (Buzsaki, 1996). The modifications in cortical activity observed in BC patients raise the question of the impact of such sleep changes on memory consolidation. Previous studies revealed an association between sleep complaint and cognitive impairment in BC patients, including memory performance (Caplette-Gingras et al., 2013; Duivon et al., 2021). A recent study also revealed that worse cognitive functioning was predicted by poorer subjective sleep quality, less robust circadian rhythms, and longer naps but not by sleep parameters (e.g., total sleep time and wake after sleep onset) assessed by actigraphy (Ancoli-Israel et al., 2022). However, none of them evaluated specifically sleep-dependent memory consolidation in BC patients.

Among other types of memory, prospective memory (PM) is of particular interest in BC because it is essential for daily living tasks, medical adherence, and autonomy. Moreover, PM difficulties have been highlighted in this population (Cheng et al., 2017; Paquet et al., 2018; Li et al., 2020). The few studies conducted mostly focused on the impact of chemotherapy and hormone receptor (Cheng et al., 2017; Li et al., 2017, 2020) and revealed difficulties in remembering *event-based* (EB) intentions. Two kinds of intentions are classically distinguished in laboratory tasks: EB intentions when the action has to be performed in response to an external event, and *time-based* (TB) ones when the action has to be executed at a specific time (Einstein and McDaniel, 1990). In addition, two components (prospective and retrospective) are fundamental to correctly execute delayed intentions (Einstein and McDaniel, 1990). The prospective component involves remembering that something has to be done at the appropriate time. The retrospective component refers to the content of the intention (i.e., remembering what has to be done).

Previous studies revealed that sleep facilitates the spontaneous remembering of intentions at the appropriate time (Scullin and McDaniel, 2010; Diekelmann et al., 2013a). Especially, sleep reinforces the association between prospective and retrospective components, whatever the type of intentions (i.e., TB or EB; Diekelmann et al., 2013b; Esposito et al., 2015). The beneficial effect of sleep on PM depends on several factors and a recent meta-analysis revealed that age and study type (i.e., experimental or observational) moderated this effect (Leong et al., 2019a). The effect size of sleep benefits is small in observational studies or studies including older adults, whereas it is generally moderate in experimental studies or studies including younger adults. Thus, one study reported that frontal slow wave activity was associated with PM in healthy adults, but this association was no longer significant when adjusted for age (Scullin et al., 2019). In return, this study revealed that REM sleep stage mediated the effect of age on sleep-dependent consolidation of PM.

In this context, this study aimed at assessing sleep-dependent consolidation of intentions in PM in BC patients. To do so, we recruited BC patients not treated with chemotherapy and HC who completed sleep questionnaires and underwent ambulatory PSG. PM was assessed using an original virtual reality based task adapted from Rehel et al. (2019). Virtual reality recreates naturalistic situations from daily life while maintaining experimental rigor and was therefore used to improve the ecological validity of the PM task. Fatigue, well-being, and psychological distress were also assessed using validated questionnaires. We hypothesized that sleep-dependent consolidation of intentions would be impaired in BC patients, and associated with changes in slow waves and sleep spindles. We also expected that poorer subjective sleep quality in patients would be associated with poorer PM performance, and that these two parameters would also be associated with greater fatigue, psychological distress and poorer well-being.

## Materials and methods

### Participants

All participants were recruited between January 2018 and March 2020 and provided written informed consent after detailed information about the study. The study was approved by the ethics committee (CPP Ile de France III; n°ID-RCB: 2017-A02778-45).

Patients’ inclusion criteria were: (i) be less than 70 years old, (ii) no metastatic BC, (iii) already undergone surgical or radiotherapy treatment, (iv) radiotherapy finished since at least 6 months and no chemotherapy treatment, (v) menopausal status since at least 1 year at the time of inclusion, (vi) no personality disorder and progressive psychiatric disorder (vii) no neurological sequelae, (viii) no drug use or alcohol abuse, (ix) be a native French speaker, (x) have at least 7 years of education.

Inclusion criteria for HC were the same as for patients, including no history of cancer and a score superior to 25 at the Montreal Cognitive Assessment (Nasreddine et al., 2005); see Duivon et al. (2018) for further details on study protocol and neuropsychological tests used.

Fifty patients, i.e., 25 patients treated with endocrine therapy (ET) and 25 patients not treated with ET approached in the French regional cancer center François Baclesse (Caen, Normandy) agreed to participate. The rationale for the target sample size was calculated according to the literature on cognitive decline in BC patients under ET (detailed in Duivon et al., 2018). Thirteen patients did not complete the entire protocol: nine withdrew, three presented symptoms similar to motion sickness during the virtual reality task, and one did not perform a session due to a technical problem with the virtual reality task. Five PSG recordings of patients were not exploitable and thus excluded from analyses. Among the twenty-five HC recruited, three withdrew and one could not stand virtual reality. Finally, 21 HC and 32 patients completed the entire protocol and were included in the analyses.

### Procedure

The experimental procedure has been fully described in Duivon et al. (2018). Only the main points are presented here. The study included three sessions with a 1-week delay in-between to minimize the risk of interference. The first session consisted of a familiarization of participants with the virtual environment and the PM task. During this session, participants were asked to memorize nine intentions and recall them 10 min after their encoding. During the second and third sessions, intentions were encoded in the morning and retrieved in the evening for the wake session, and encoded in the evening and retrieved the next morning for the sleep session. The order of sleep and wake sessions was counterbalanced between participants. During the wake session, participants performed their usual activities, with the instruction not to nap. During the sleep session, participants went back home or spent the night in a hotel. Sleep was monitored using ambulatory PSG.

### Polysomnography

Participants underwent a PSG using a portable device (Siesta, Compumedics, Victoria, Australia). The PSG consisted in recording the electroencephalogram (EEG), electrooculogram (EOG), electrocardiogram (ECG), chin electromyogram (EMG), respiratory movements using thoracic and abdominal belts, respiratory airflow using nasal and oral thermistors, and oxygen saturation using a finger pulse oximeter. For the EEG recording, twenty electrodes were placed over the scalp according to the international 10–20 system (Fp1, Fp2, F3, F4, F7, F8, Fz, C3, C4, Cz, T3, T4, P3, P4, Pz, O1, O2, vertex ground, and a bi-mastoid reference), with impedances kept below 5 kΩ. The EEG signal was digitalized at a sampling rate of 256 Hz, high-pass (0.3 Hz) and low-pass (35 Hz) filters were applied for the visual scoring. Sleep stages (N1, N2, N3, and REM) were visually scored by an electrophysiology technician (S.R.) in epochs of 30 s according to the AASM rules (Berry et al., 2017). Standard sleep parameters, including the percentage of each sleep stage, as well as respiratory parameters, such as the apnea-hypopnea index (AHI), were computed. Artifacts (eye movements, ECG, EMG, or movement-related artifacts) were detected visually and rejected (J.P.), and analyses were conducted on artifact-free epochs. Automatic quantification of sleep spindles and slow waves from the Cz-Pz derivation was performed during N2-N3 and N3 stages respectively, using the ASEEGA software (PHYSIP, Paris, France). The validation of the original algorithm is fully described elsewhere (Berthomier et al., 2007). An adapted amplitude slow wave’s criterion (Rosinvil et al., 2021) instead of the 75°microVolts standard criterion was used to detect slow waves. More specifically, the detection method for spindles and slow waves was based on data-driven criteria using multiple iterations in order to cope with inter-subject and inter-recording variability. The first iteration determined the recording-specific thresholds on the basis of EEG amplitude and EEG power ratios in frequency bands. The second iteration determined the precise temporal localization of each event. The final iteration validated the pre-detected events according to frequency and duration criteria (> 0.5 s) for spindles (Dang-Vu et al., 2017), amplitude and duration criteria (0.25 s < duration < 2 s) for slow waves. For spindles, iteration 1 and 3 dealt with raw EEG data, while iteration 2 was applied on the EEG filtered in the spindle (sigma) frequency range. For the slow waves, iteration 1 dealt with raw EEG data, while iterations 2 and 3 were applied on the EEG filtered in the slow waves (delta) frequency range. Finally, the density of spindles (i.e., number of spindles during stages N2-N3 divided by the time spent in N2-N3) and slow waves (i.e., number of slow waves during stage N3 divided by the time spent in N3) was computed.

### Questionnaires

Subjective sleep disturbances were assessed using the Pittsburgh Sleep Quality Index (PSQI; Buysse et al., 1989) a 19-item questionnaire measuring sleep habits and disturbances over the previous month (total score ranging from 0 to 21). In cancer population, a PSQI score above 8 is considered to reflect poor sleep (Carpenter and Andrykowski, 1998), instead of five in healthy population (Buysse et al., 1989). Insomnia symptoms were measured with the Insomnia Severity Index (ISI; Morin et al., 2011) a seven-item questionnaire (score ranging from 0 to 28). The cut-off score of eight indicating a subthreshold insomnia has been validated in cancer patients (Savard et al., 2005; Michaud et al., 2021). For both questionnaires, higher scores indicate higher sleep disturbances and insomnia related symptoms.

The level of alertness/sleepiness was measured with the Karolinska Sleepiness Score (KSS, Akerstedt and Gillberg, 1990) before every encoding and retrieval phases of the PM task. KSS is a nine-item questionnaire (total score ranging from 1 to 9) with higher scores indicating greater sleepiness.

Depressive symptoms were assessed with the Beck Depression Inventory (BDI), a validated 13-item questionnaire with a total score ranging from 0 to 39 (Beck et al., 1961). Anxiety was assessed with the State Trait Anxiety Inventory (Spielberger et al., 1970). The first 20 items assess state anxiety (STAI-A), i.e., how the participant feels at the moment of the assessment; the remaining 20 items assess trait anxiety (STAI-B), i.e., how the participant usually feels; each range from 20 to 80. The STAI questionnaire was fulfilled during the first session of familiarization, thus the STAI-A score was not representative of the anxiety state at the time of sleep session and only the STAI-B score was reported here. For both questionnaires, i.e., BDI and STAI-B, higher values indicate higher depression and trait-anxiety symptoms.

Fatigue and well-being were assessed in BC patients only, using the Functional Assessment of Chronic Illness Therapy–Fatigue (FACIT-F; Yellen et al., 1997), a 13-item questionnaire (score ranging from 0 to 52) and the Functional Assessment of Cancer Therapy–General (FACT-G; Cella et al., 1993), a 27-item questionnaire (score ranging from 0 to 108). For both questionnaires, higher scores indicate lower fatigue and greater well-being, respectively.

### Prospective memory task

The PM task was adapted from Rehel et al. (2019).

During the encoding phase, participants were asked to learn nine intentions that they had to remember after a certain amount of time (recall time specified by the session). The nine intentions included three TB intentions (e.g., “at 12:11, go to the restaurant for lunch”), and six EB intentions (e.g., “at the cafeteria, buy a black coffee” or “at the child-care center, ask for a map of the Memorial”). A new set of nine intentions was learnt in every session. Each intention was presented on a computer screen, then a cued-recall test was performed and repeated until each intention was correctly learnt. Finally, to ensure that all intentions were correctly encoded, they were retrieved in a last global cued-recall test.

During the retrieval phase, participants were immersed in the virtual environment for a maximum of 20 min. The environment featured a reproduction of the Memorial museum dedicated to World War II and located in Caen (Normandy, France). Participants were placed in an immersive room composed of four wide screens for 3D stereoscopic projections, wore stereoscopic glasses with position sensors and freely navigated in the museum using a joystick. Participants were asked to visit the museum while memorizing pictures displayed in the environment (ongoing task) and recalling intentions at the appropriate place or time. EB intentions had to be recalled in response to a specific cue in the environment, and TB intentions at the appropriate time. For this purpose, a button on the joystick was available to check time.

Each intention was rated according to three components, i.e., the prospective component, the retrospective component, and the associative one (correct action recalled at the appropriate time or place). A maximum of two points was awarded to every component for a correct recall i.e., if every detail of the retrospective component was recalled at the appropriate time. If the intention was recalled during the second passage or within 1 min of the target time, only one point was attributed to the prospective score. If the intention was recalled at a later passage or within 2 min of the target time, only 0.5 points were awarded to the prospective component [see Duivon et al. (2018) for scoring details]. Each intention had therefore a maximum score of six points corresponding to the sum of the three components scores. Thus, each type of intention had a maximum score of 36 for EB intentions (6*6 EB intentions) and 18 for TB intentions (6*3 TB intentions). Every component had a maximum score of 18 corresponding to the sum of component scores for each intention (2*9 intentions).

### Statistical analyses

Analyses were performed using the R software (version 4.0.3), with statistical significance set at *p* < 0.05. Normality hypothesis was assessed using the Shapiro test. When the normality hypothesis was rejected, data were analyzed using non-parametric tests.

Wilcoxon tests were performed to compare demographic characteristics, scores of depression (BDI), scores of sleep questionnaires (ISI, PSQI), score of alertness/sleepiness (KSS), and encoding performance (i.e., number of intentions retrieved during the last global cued recall test). The STAI-B scores were compared using student *t* test.

Analyses of covariance (ANCOVA) were conducted with sleep architecture parameters (i.e., general sleep parameters and sleep stages) as dependent variables, group as independent variable, and the Apnea-Hypopnea index (AHI) as covariate. As no significant effect of AHI was observed on quantification of slow waves and sleep spindles, comparisons between groups were realized with Wilcoxon tests.

An analysis of variance (ANOVA) was performed to evaluate the time-of-day effect on PM performance with PM scores at session 1 as the dependent variable, group and session-time (morning or evening) as independent variables. A paired ANOVA was performed to evaluate group and session effect on PM retrieval using PM scores as the dependent variable, group and session (wake and sleep) as independent variables. Furthermore, in order to evaluate PM consolidation during sleep (PM scores at the sleep session) accounting for performance obtained during the day (PM scores at the wake session), we performed an ANCOVA for each PM score. PM performance during the sleep session was the dependent variable, group the independent variable, and PM performance during the wake session the covariate. As the normality hypothesis was rejected for the retrospective PM score, this latter was log transformed.

In order to control for the effect of ET received by some patients, the analyses of sleep questionnaires, PSG parameters (architecture, slow waves, and spindles characteristics) and PM scores detailed above were conducted with ET status (i.e., with three groups: the patients treated with ET, the patients not treated with ET and HC) as covariate. When the normality hypothesis was rejected, the score was log transformed.

Then, Spearman correlation analyses were performed in the whole sample of participants (HC and BC patients), to assess the associations between subjective sleep assessments (PSQI and ISI scores), PM scores (each intention and component) during the sleep session, psychological distress (BDI and STAI-B scores) and well-being and fatigue (FACT-G and FACIT-F, in BC patients only). Finally, Spearman correlation analyses were performed to test the associations between PSG parameters (i.e., % of N3 and REM sleep, number of awakenings > 1 min, spindles and slow waves parameters) and PM scores (each intention and component) during the sleep session.

## Results

### Participants characteristics

Demographic characteristics of participants and clinical characteristics of patients are reported Table 1. No significant difference on demographic characteristics and levels of anxiety and depression was observed between groups (all *ps* > 0.40).

**TABLE 1:** Demographic and clinical characteristics of participants (mean ± SD).

Demographic characteristics	HC (*n* = 21)	BC (*n* = 32)	*P*-values
Age (years)	62.6 ± 4.4	61.8 ± 5.2	0.63
Education (years)	11.8 ± 1.7	11.8 ± 3.5	0.44
Anxiety (STAI-B)	42.3 ± 9.3	40.8 ± 10.5	0.60
Depression (BDI)	4.1 ± 3.2	3.6 ± 3.2	0.44
**Clinical characteristics**	**HC (*n* = 21)**	**BC (*n* = 32)**	
Stage of the cancer, n (%): 0 I IIA	NA	17 (53%) 12 (38%) 3 (9%)	
Tumorectomy, n (%) Mastectomy, n (%)	NA	29 (91%) 4 (13%)*	
Time since radiotherapy (months)	NA	8 ± 2.4	
Treated with endocrine therapy, Not treated with endocrine therapy	NA	16 (50%) 16 (50%)	
Fatigue (FACIT-F)	NA	36.6 ± 10	
Well-being (FACT-G)	NA	84.6 ± 17	

*One patient receiving tumorectomy and mastectomy.

HC, healthy controls; BC, breast cancer; STAI-B, State Trait Anxiety Inventory–Trait; BDI, Beck depression inventory; FACIT-F, Functional Assessment of Chronic Illness Therapy–Fatigue; FACT-G, Functional Assessment of Cancer Therapy–General.

Age, Education, and Depression were compared with Wilcoxon tests and Anxiety with the student’s *t*.test.

### Sleep

The analyses of sleep questionnaires revealed a significant difference between groups on subjective sleep disturbances (PSQI, *p* = 0.020) and insomnia severity (ISI, *p* = 0.004), indicating that BC patients had greater sleep complaints and more severe insomnia symptoms than HC (Table 2). This difference between BC and HC remained significant when ET status (i.e., the three groups: patients receiving ET, patients not receiving ET and HC) was added as covariate for the ISI but not the PSQI (see Supplementary Table 1).

**TABLE 2:** Sleep characteristics, i.e., subjective sleep quality and sleep architecture (mean ± SD).

Sleep questionnaires	HC (*n* = 21)	BC (*n* = 32)	*P*-values		
Sleep disturbances (PSQI total score)	5.48 ± 2.8	8.29 ± 4.5	**0.020**		
Insomnia severity index (ISI score)	7.19 ± 4.5	11.9 ± 6.6	**0.004**		
**Sleep architecture (AHI as co-variate)**	**HC (*n* = 21)**	**BC (*n* = 32)**	***F (1,50)***	***P*-values**	**η^2^**
Total sleep time (min)	356 ± 68	355 ± 68	0.001	0.97	< 0.01
Sleep efficiency (%)	76.9 ± 11	74.1 ± 11	0.75	0.39	0.01
Number of awakenings > 1 min	6.76 ± 2.8	9.59 ± 4.4	7.10	**0.010**	0.12
WASO%	16.5 ± 12	19.9 ± 11	1.22	0.28	0.02
N1 (% TST)	8.6 ± 3.7	8.86 ± 5.2	0.059	0.81	< 0.01
N2 (% TST)	50.3 ± 7.2	52.0 ± 6.0	0.91	0.35	0.02
N3 (% TST)	22.9 ± 6.1	22.6 ± 7.3	0.024	0.88	< 0.01
REM (% TST)	18.3 ± 4.8	16.6 ± 3.9	1.95	0.17	0.04
Apnea-hypopnea index (AHI)	19.6 ± 13	19.8 ± 12	NA	0.83	NA

HC, Healthy Controls; BC, breast cancer patients; PSQI, Pittsburgh Sleep Quality Index; ISI, Insomnia Severity Index; WASO, Wake After Sleep Onset; TST, Total sleep time; REM, Rapid-Eye-Movement sleep. *P*-values < 0.05 are in bold. Effects size: Small (η^2^ ≥ 0.01), medium (η^2^ ≥ 0.06), and large (η^2^ ≥ 0.14). Comparison of the PSQI and the AHI scores were realized with Wilcoxon tests and ISI score with the student’s t.test. Comparison of the sleep architecture was realized with analyses of co-variance with AHI as covariate.

Subjective sleep disturbances and insomnia severity were positively associated with trait anxiety assessed using the STAI-B (PSQI: *r* = 0.28, *p* = 0.043; ISI: *r* = 0.31, *p* = 0.027) in the whole sample of participants. The insomnia severity was positively associated with depression assessed using the BDI (ISI: *r* = 0.35, *p* = 0.01). Thus, higher sleep complaints were related with greater psychological distress in the whole group of participants. PSQI and ISI scores were also negatively associated with FACIT-F (PSQI: *r* = −0.59, *p* < 0.001; ISI: *r* = −0.51, *p* = 0.003) and FACT-G scores (PSQI: *r* = −0.65, *p* < 0.001; ISI: *r* = −0.57, *p* < 0.001) in BC patients. Thus, higher sleep complaints were related to greater fatigue and poorer well-being in BC patients (see Supplementary Table 5).

Standard PSG parameters of the two groups are reported in Table 2. A significant effect of group [*F*(1,50) = 7.10, *p* = 0.010] was observed only for the number of awakenings longer than 1 min, with patients having significantly more awakenings than HC.

Sleep spindles frequency differed between groups (*p* = 0.025) with patients having a faster frequency than HC (Table 3). The peak-to-peak amplitude of slow waves also differed between groups (*p* = 0.040), with lower amplitude in BC patients compared with HC.

**TABLE 3:** Sleep spindles features during N2 + N3, and slow waves features during N3 (mean ± SD).

Parameters		HC (*n* = 21)	BC (*n* = 32)	*P*-values
Spindles (N2 + N3)	Frequency (Hz)	13.6 ± 0.6	13.9 ± 0.5	**0.025**
	Maximum amplitude (μV)	10.0 ± 2.5	9.57 ± 2.7	0.41
	Density (number per epoch)	2.81 ± 0.9	2.79 ± 1.4	0.55
Slow waves (N3)	Peak to peak amplitude (μV)	80.4 ± 17	72.6 ± 20	**0.040**
	Density (number per epoch)	9.01 ± 3.6	7.95 ± 3.9	0.13

HC, Healthy Controls; BC, breast cancer patients. *P*-values < 0.05 are in bold. Analyses were realized with Wilcoxon tests.

For the sake of completeness, we conducted all the analyses on PSG parameters detailed above adding the ET status as covariate. These analyses revealed that the effect of group on the number of awakenings longer than 1 min, the spindle frequency and slow waves amplitude was no longer significant (see Supplementary Table 1). On the contrary, a difference on slow waves density became significant [*F*(1,50) = 4.91, *p* = 0.031].

### Prospective memory performance

#### Effect of time-of-day on prospective memory performance

In each group, half of the participants performed the session 1 in the morning and the other half in the evening in order to control for an effect of time-of-day on PM performance. The ANOVA revealed no significant effect of time-of-day [*F*(1,49) = 0.11, *p* = 0.74] and group [*F*(1,49) = 0.20, *p* = 0.66], and no significant interaction between these factors [*F*(1,49) = 0.35, *p* = 0.56] on encoding performance. No significant effect of time-of-day [*F*(1,49) = 0.05, *p* = 0.82] and group [*F*(1,49) = 0.50, *p* = 0.48], and no significant interaction between these factors [*F*(1,49) = 0.12, *p* = 0.74] on global PM performance (i.e., sum of three components scores) was observed. Moreover, no significant effect of group, time-of-day and interaction were observed whatever the type of intention and component (all *p values* > 0.05, results not shown).

A comparison of the level of alertness/sleepiness (KSS) was performed between groups. Whether at the wake session or the sleep session, or before encoding or retrieval, no significant difference was observed between the groups (all *p values* > 0.2, results not shown).

These results indicate that there was no different effect of time-of-day on encoding and recall performance between groups.

#### Encoding

The number of intentions retrieved during the last cued-recall test of the encoding phase did not differ significantly between groups (mean for the sleep session HC = 8.2; BC = 7.7; *p* = 0.25; mean for the wake session HC = 8.1; BC = 7.9; *p* = 0.75). This effect became significant when ET status was added as covariate for both sessions (all *p value*s < 0.02).

#### Effect of group and session (sleep vs. wake) on prospective memory performance

An analysis of variance revealed better global PM performance during the sleep session compared to the wake session [*F*(1,51) = 21.5, *p* < 0.001]. Neither a significant effect of group [*F*(1,51) = 1.48, *p* = 0.23], nor a significant interaction between group and session [*F*(1,51) = 0.09, *p* = 0.76] was observed. Similar results were observed for each intention and component (all *p values* > 0.15, see Table 4 for further details). Thus, better PM retrieval performance was observed after a night of sleep rather than after an equivalent period of daytime wakefulness, in both groups of participants.

**TABLE 4:** Prospective memory (PM) performances during the wake and sleep sessions (mean ± SD) and analyses of the group and session effects.

	HC (*n* = 21)		BC (*n* = 32)		Group effect		Session effect		Group effect (accounting for wake session performance)		
	Wake	Sleep	Wake	Sleep	*F (1,51)*	*P*-values	*F (1,51)*	*P*-values	*F (1,49)*	*P*-values	η^2^
**Total (/54)**	30.0 ± 7.7	34.8 ± 8.0	26.6 ± 10	32.2 ± 11	1.48	0.23	21.5	**< 0.001**	0.05	0.83	< 0.01
**Intentions**											
EB (/36)	22.4 ± 6.2	25.5 ± 6.3	20.2 ± 7.8	24.3 ± 7.1	1.07	0.31	14.0	**< 0.001**	0.02	0.89	< 0.01
TB (/18)	7.52 ± 3.0	9.24 ± 4.6	6.45 ± 4.6	7.88 ± 5.0	1.27	0.27	6.21	**0.016**	0.36	0.55	< 0.01
**Components (/18)**											
Prospective	8.86 ± 3.2	10.7 ± 3.6	8.36 ± 3.5	9.56 ± 3.6	0.96	0.33	9.37	**0.004**	1.03	0.32	< 0.01
Retrospective	12.81 ± 2.7	13.8 ± 3.1	11.3 ± 4.1	13.5 ± 3.5	1.04	0.31	15.1	**< 0.001**	0.30	0.59	< 0.01
Associative	8.29 ± 3.4	10.3 ± 2.8	7.00 ± 3.9	9.06 ± 4.3	1.77	0.19	19.1	**< 0.001**	0.25	0.62	< 0.01

HC, healthy controls; EB, event-based; *P*-values < 0.05 are in bold. TB, time-based. Analyses of variance were used for the group and session effect. Analyses of co-variance were used with performance during the wake session as co-variate for the group effect (accounting for wake session performance).

#### Sleep-dependent prospective memory retrieval

Table 4 summarizes PM performance in BC patients and HC during the wake and sleep sessions. Analyses of co-variance were conducted on PM scores obtained during the sleep session, accounting for PM scores obtained during the wake session. PM performance did not differ between groups whatever the type of intention (EB or TB; all *p values* > 0.55) or the component (Prospective, Retrospective, and Associative; all *p values* > 0.39). When ET status was added as covariate, a significant difference between BC and HC was observed for the prospective [*F*(1,49) = 5.3, *p* = 0.025] and the associative components [*F*(1,49) = 4.50, *p* = 0.039], with patients having lower scores than HC.

Prospective memory performance was not significantly related with psychological distress, i.e., STAI-B and BDI scores in the whole sample of participants (see Supplementary Table 5). A significant correlation was observed between the retrieval of the retrospective component and the FACIT-F score (*r* = 0.38, *p* = 0,030). Thus, higher fatigue is related with lower retrieval of the retrospective component in BC patients. No other significant correlation was observed.

#### Associations between sleep and prospective memory performance

No significant correlation was observed during both sessions between encoding performance (i.e., number of intentions retrieved during the last cued-recall test of the encoding phase), scores on sleep questionnaires (PSQI, ISI, all *p values* > 0.1, results not shown) and fatigue (FACIT-F only in BC patients, all *p values* > 0.1).

The PSQI total score was significantly associated with the retrieval of EB intentions (*r* = −0.36, *p* = 0.010) and retrospective components of the whole intentions (*r* = −0.29, *p* = 0.038). The ISI score was significantly associated with the retrieval of EB intentions (*r* = −0.41, *p* = 0.003) and retrospective components of the whole intentions (*r* = −0.42, *p* = 0.002). Thus, higher sleep disturbances and insomnia complaints were associated with poorer recall of EB intentions and retrospective components after a night of sleep in the whole sample of participants. Furthermore, FACIT-F score was significantly related with the retrospective component score (*r* = 0.38, *p* = 0.03), indicating that greater fatigue was related with poorer recall of the retrospective component of intentions in BC patients.

No significant correlation was observed between PSG parameters (% of N3 and REM sleep, number of awakenings longer than 1 min and all spindles and slow waves parameters) and PM performance (all *p values* > 0.05, see Supplementary Table 4).

## Discussion

The aim of this study was to assess, sleep-dependent consolidation of intentions in PM in BC patients not receiving chemotherapy. First, BC patients reported greater sleep disturbances and insomnia symptoms than HC. Greater sleep complaints were related with poorer PM performance and markers of quality of life, i.e., fatigue, well-being and psychological distress. An objective assessment of sleep using PSG revealed more awakenings longer than 1 min, decreased amplitude of slow waves and increased frequency of sleep spindles in BC patients compared to HC. These subtle sleep modifications were not related to sleep-dependent consolidation of intentions whose remembering did not differ between groups.

Greater subjective sleep disturbances and insomnia severity were related to greater difficulty remembering EB intentions and retrospective components in the whole group of participants. These results are in line with previous studies in BC patients revealing a significant correlation between sleep complaint and cognitive performance (Duivon et al., 2021) including memory performance (Caplette-Gingras et al., 2013). In the present study, both groups had greater PM performance during the sleep session than during the wake session, highlighting the significant benefit of sleep on consolidation of intentions. BC patients had no encoding difficulties as evidenced by the equivalent number of intentions recalled at the end of the encoding phase, except when controlling for ET. Moreover, encoding performance was not related to subjective sleep assessment and fatigue, meaning that self-reported sleep disturbances and fatigue could have a specific impact on retrieval process but not on memory encoding. According to the Multiprocess framework (McDaniel and Einstein, 2000), PM retrieval relies on a continuum from strategic processes to relatively spontaneous processes depending on several characteristics such as the distinctiveness of the cue and the strength of the association between the prospective and retrospective components. Previous studies conducted in healthy subjects, revealed that sleep benefits spontaneous retrieval processes, by strengthening the association between the two components, rather than strategic monitoring processes (Diekelmann et al., 2013b; Esposito et al., 2015). In our study, no significant correlation was found with the associative component to confirm this hypothesis. Nevertheless, the correlation with the recall of EB intentions and the retrospective component suggests a benefit of sleep on the episodic memory dimension, i.e., the spontaneous processes of remembering the corresponding action when the cue is detected, rather than on the strategic processes needed to check the environment and estimate time. However, contrary to our hypotheses, sleep-dependent consolidation of intentions in PM was not impaired in BC patients. In the present study, the mean score of PSQI of BC patients was equal to the cutoff score proposed in this pathology (Carpenter and Andrykowski, 1998). Moreover, the mean ISI score in BC patients was considered to reflect subthreshold insomnia (Morin et al., 2011). Thus, although BC patients had greater sleep complaints than HC related to poorer PM performance, the level of subjective sleep disturbances in this group of patients was relatively low. This may explain the lack of sleep-related impairment of PM consolidation in BC patients.

Subtle sleep changes were observed when analyzing PSG data. BC patients had more awakenings longer than 1 min than HC, but neither sleep efficiency nor the time awake after sleep onset differed between groups. Thus, these awakenings did not modify sleep architecture of BC patients. Concerning PM, SWS appears to be involved in the consolidation of intentions in young adults (Diekelmann et al., 2013a; Leong et al., 2019b,^2021^). However, these results are not consistently reported since Cunningham et al. (2021) found a negative association between SWS and PM performance in young adults. In the study by Leong et al. (2021), while older adults had lower PM performance and spent less time in SWS than young adults, no significant relation was found between SWS and PM in the older group. The authors suggested that the role of SWS on PM consolidation may be disrupted and thus ineffective in older adults. Meanwhile, Scullin et al. (2019), in a study conducted in adults aged 18–84 years, revealed that REM sleep duration explained the variance in PM consolidation when controlling for age. In the present study, in which we included older participants, neither time spent in SWS (N3 sleep) nor time spent in REM sleep were related to PM performance. Further analyses on REM sleep, which would rather be involved in intentions consolidation in older adults, should be conducted. Distinguishing for instance between phasic and tonic phases (Simor et al., 2020), which might play distinct roles in memory consolidation, would be interesting.

Aging is characterized by a decrease in the amplitude and density of slow waves and density and duration of sleep spindles (Mander et al., 2017). In a study assessing sleep-dependent consolidation of intentions in young and older adults, Scullin et al. (2019) reported a correlation between slow wave activity and the recall of EB intentions, which was no longer significant when adjusted for age. As suggested for time spent in SWS, the role of slow waves on sleep-dependent memory consolidation must be less effective in older adults. This hypothesis could explain the lack of relationship between slow waves characteristics and PM performance, and the lack of PM difficulties despite lower slow wave’s amplitude in BC patients. In the present study, only a change in slow wave’s amplitude was observed. Thus, we could surmise that the density of slow waves, rather than their amplitude, is relevant for memory consolidation but this hypothesis requires further investigations. A significant increase in spindle frequency was also observed in BC patients but this change was not related to PM performance. The lack of significant association could be explained by the low variability of this spindle feature. However, this result is consistent with previous studies conducted in healthy adults and revealing no significant association between sleep spindles and retrieval of intentions (Scullin et al., 2019; Leong et al., 2021).

Prospective memory remembering was related to subjective sleep disturbances but not to objective sleep changes. These results are in line with a recent study in BC revealing a relationship between neuropsychological performance and subjective sleep quality but not with objective sleep parameters measured with actigraphy (Ancoli-Israel et al., 2022). The discrepancy between subjective and objective sleep measures frequently observed in healthy subjects (Rezaie et al., 2018) is also observed in BC patients. Reinsel et al. (2015) showed no difference in sleep architecture between patients with and without insomnia complaints. In the same way, a discrepancy is frequently observed between subjective and objective measures of cognitive functioning in BC patients (Ganz et al., 2013; Bray et al., 2018). Some studies suggest that this discrepancy could be due to the nature of the assessment. PSG and neuropsychological tests have a poor ecological validity which may underestimate difficulties measured with questionnaires (Savard and Ganz, 2016). In the present study, we used a virtual reality-based task to reproduce as finely as possible situations of everyday life. This type of paradigm is supposed to be more sensitive to measure cognitive difficulties experienced in everyday life (Lecouvey et al., 2017; Rehel et al., 2019). Nevertheless, PM impairment in the group of BC patients might be too subtle to be detected even by an ecological task but still related with worse subjective sleep quality and fatigue. This result is reminiscent of the study by Mihuta et al. (2016) who reported no PM deficit in BC patients using a virtual reality task but reported associations between PM scores and cognitive complaints. The laboratory context encourages participants to muster all their abilities to succeed in the task and the ongoing task is not as demanding as in real-world situations. These limitations, compared to an assessment with a naturalistic task, seem difficult to overcome. However, our PM task could be made even more ecological. We could take the example of the Virtual Reality Everyday Assessment lab (VR-EAL) where realistic intentions were used such as “Take the chocolate pie out of the oven” and “Collect the carrot cake from the bakery at 12 pm” (Kourtesis et al., 2020).

Fatigue, psychological distress, sleep disturbances, and cognitive impairment are the most common side effects reported by BC patients (Bower, 2008). In the study by Xu et al. (2018), using a Bayesian network method to represent multivariate relationships between sets of variables, cognitive performance was directly related to sleep complaints, and also indirectly related to fatigue (through sleep) and depression (through sleep and fatigue). Higher fatigue has been previously related with PM impairment in BC patients (Paquet et al., 2013). In our study, worse PM performance was related to poorer sleep quality and greater fatigue and in turn sleep disturbances were associated with worse psychological distress, fatigue, and well-being. Thus, we can surmise that PM impairment usually reported in BC patients (not treated with chemotherapy) would rather be related to several factors affecting their quality of life such as greater sleep disturbances, fatigue, and psychological distress, rather than to a specific alteration of physiological mechanisms involved in sleep-dependent memory consolidation. As the patients included in this study had no major psychological distress in comparison to HC, and no major sleep disturbances, fatigue or poorer well-being (according to the normative data of the questionnaires), this could explain the lack of PM impairment in BC patients. Further studies with larger samples are needed to better understand the role of factors related to quality of life on sleep-dependent memory consolidation in BC patients not treated with chemotherapy.

The patients included in this study were in the early stages of BC (0, I, and II), not treated with chemotherapy and assessed at least 6 months after radiation therapy. These criteria were chosen to minimize as much as possible the negative effect of the treatments, particularly the well-known effects of chemotherapy on sleep and memory. However, half of BC patients were under ET at the time of assessment and ET has been associated with higher sleep complaints (Dhruva et al., 2012) and lower episodic memory performance (Underwood et al., 2018, 2019). Thus, in order to control for the impact of ET on sleep-dependent PM consolidation, complementary analyses were performed, adding the ET status as covariate. These analyses revealed that there was no longer a significant difference for the numbers of awakenings longer than one minute, the frequency of spindles and the amplitude of slow waves between BC and HC. Nevertheless, a significant difference was revealed for the density of slow waves, the encoding of new intentions and the retrieval of prospective and associative components. Thus, it appears that ET had subtle effect on objective sleep parameters and PM functioning. Further investigations with larger sample size should be conducted to precisely address this issue.

## Conclusion

This study reveals greater sleep complaints and subtle sleep changes in BC patients compared to HC. Despite the role of slow waves and spindles in sleep-dependent memory consolidation, the modifications observed in BC patients had no significant impact on sleep-dependent PM consolidation. Our results suggest that PM impairment reported in previous studies would be due more to an altered quality of life reflected by several factors such as poorer sleep quality and fatigue, rather than to a slight alteration of sleep-dependent memory consolidation mechanisms. Thus, in this study focusing on patients at early stages of BC, not treated with chemotherapy and without psychological distress and severe sleep disturbances, sleep-dependent consolidation of intentions was not impaired.

## Data availability statement

The original contributions presented in this study are included in the article/Supplementary material, further inquiries can be directed to the corresponding author.

## Ethics statement

The studies involving human participants were reviewed and approved by CPP Ile de France III (n°ID-RCB: 2017-A02778-45). The patients/participants provided their written informed consent to participate in this study.

## Author contributions

MD collected and analyzed the data and wrote the first draft of the manuscript. SR, CB, and JP realized sleep analyses. CS-D, JG, GE, CL, and FV participated in the investigation. J-MG, BC, BG, and JP participated in the project administration. BG, JP, BD, FE, and FJ participated in the funding acquisition and design of the study. BG, BD, GR, and JP participated to the rewriting and editing of the study. All authors have read and agreed to the published version of the manuscript.

## Abbreviations

AHI: Apnea-Hypopnea Index
BC: Breast cancer
EB: Event-based
ET: Endocrine therapy
HC: Healthy control
PM: Prospective memory
REM: Rapid eye movement
TB: Time-based
TST: Total sleep Time
WASO: Wake after sleep onset.
