# Supplementary material for: Sleep-dependent memory consolidation in breast cancer: Use of a virtual reality prospective memory task
Source: Front Neurosci. 2022 Sep 7;16:908268. doi: 10.3389/fnins.2022.908268 (PMC9489900; doi:10.3389/fnins.2022.908268)
Supplement: Supplementary file 1 [file Table_1.DOCX]

Supplementary Table 1. Analyses of sleep characteristics, i.e. subjective sleep quality and sleep architecture with ET status as covariate

| Sleep questionnaires | F (1,49) Group effect | *p*-values | *η^2^* | F (1,49)  ET Status | *p*-values |
| --- | --- | --- | --- | --- | --- |
| Sleep disturbances (PSQI total score) log | 2.42 | 0.13 | 0.04 | 3.70 | 0.060 |
| Insomnia Severity Index (ISI score) | 5.16 | **0.028** | 0.09 | 1.12 | 0.30 |
| Sleep architecture (AHI as co-variate) | **F (1,49*)* Group effect** | ***p*-values** | ***η^2^*** | **F (1,50)**  **ET Status** | ***p*-values** |
| Total Sleep Time (min) | 0.83 | 0.37 | 0.02 | 0.28 | 0.60 |
| Sleep Efficiency (%) | <0.01 | 0.97 | <0.01 | 0.91 | 0.35 |
| Number of awakenings > 1min | 0.63 | 0.43 | 0.01 | 6.60 | **0.013** |
| WASO % | 0.04 | 0.84 | <0.01 | 1.59 | 0.21 |
| N1 (% TST) | 1.34 | 0.25 | 0.02 | 0.11 | 0.74 |
| N2 (% TST) | 0.06 | 0.80 | <0.01 | 0.87 | 0.36 |
| N3 (% TST) | 0.13 | 0.72 | <0.01 | <0.01 | 0.99 |
| REM (% TST) | 0.63 | 0.43 | 0.01 | 1.31 | 0.26 |

PSQI: Pittsburgh Sleep Quality Index; ISI: Insomnia Severity Index; AHI: Apnea-Hypopnea Index; WASO: Wake After Sleep Onset; REM: Rapid-Eye-Movement sleep. *P*-values < 0.05 are in bold**.** Effect size: Small (η2 ≥ 0.01), medium (η2 ≥ 0.06), and large (η2 ≥ 0.14). Analyses of co-variance were used with ET status as covariate for sleep questionnaires and AHI + ET status as co-variates for PSG measures.

Supplementary Table 2. Analyses of sleep spindles features during N2+N3, and slow waves features during N3 with ET status as covariate

| Parameters | | F (1,50)  Group effect | *p*-values | *η^2^* | *F (1,50)*  ET Status | *p*-values |
| --- | --- | --- | --- | --- | --- | --- |
| Spindles  (N2+N3) | Frequency (Hz) ^log^ | 0.80 | 0.38 | <0.01 | 2.34 | 0.13 |
|  | Maximum amplitude (µV) ^log^ | 1.26 | 0.27 | 0.02 | 2.14 | 0.15 |
|  | Density (number per epoch) ^log^ | 0.054 | 0.82 | <0.01 | 0.53 | 0.47 |
| Slow waves  (N3) | Peak to peak amplitude (µV) ^log^ | 0.64 | 0.43 | 0.01 | 5.75 | **0.02** |
|  | Density (number per epoch) | 4.91 | **0.03** | 0.09 | <0.01 | 0.99 |

^log^: log transformed values.

*P*-values < 0.05 are in bold. Analyses of co-variance were used with ET status as covariate.

Supplementary Table 3. Analyses of PM performance during the sleep session, accounting for performance obtained in the wake session and ET status

|  | F (1,49)  Group effect | *p*-values | *η^2^* | F (1,50)  ET Status | *p*-values |
| --- | --- | --- | --- | --- | --- |
| **Intentions** |  |  |  |  |  |
| EB (/36) | 3.56 | 0.065 | 0.05 | 0.66 | 0.42 |
| TB (/18) | 1.82 | 0.18 | 0.03 | <0.01 | 0.99 |
| **Components (/18)** |  |  |  |  |  |
| Prospective | 5.31 | **0.025** | 0.07 | <0.01 | 0.98 |
| Retrospective ^log^ | 2.07 | 0.16 | 0.03 | 1.86 | 0.18 |
| Associative | 4.50 | **0.039** | 0.05 | 0.27 | 0.60 |

^log^: log transformed values.

EB: event-based; TB: time-based. *P*-values < 0.05 are in bold. Analyses of co-variance were used with ET status as covariate.

Supplementary Table 4. Spearman correlations of PM scores with subjective and objective sleep parameters

|  | EB | TB | Prospective | Retrospective | Associative |
| --- | --- | --- | --- | --- | --- |
| PSQI | -0.36** | 0.01 | -0.13 | -0.29 * | -0.2 |
| ISI | -0.41 ** | -0.14 | -0.27 | -0.42 ** | -0.26 |
| N3 (% TST) | 0.00 | -0.05 | -0.06 | -0.08 | -0.04 |
| REM (% TST) | 0.04 | -0.07 | 0.01 | -0.05 | 0.03 |
| Number of awakenings > 1min | 0.08 | 0.09 | 0.14 | 0.12 | 0.06 |
| Density (Spindles) | -0.04 | 0.04 | -0.09 | 0.02 | 0.02 |
| Frequency (Spindles) | -0.22 | -0.12 | -0.16 | -0.17 | -0.21 |
| Amplitude (Spindles) | -0.18 | -0.09 | -0.16 | -0.22 | -0.14 |
| Density (Slow waves) | 0 | 0.09 | -0.03 | 0.11 | 0.04 |
| Amplitude (Slow waves) | -0.21 | -0.02 | -0.11 | -0.25 | -0.16 |

EB: Event-based; TB: Time-based; PSQI: Pittsburgh Sleep Quality Index; ISI: Insomnia Severity Index; REM: Rapid-Eye-Movement sleep. * *p* < 0.05; ** *p* < 0.01

Supplementary Table 5. Spearman correlations of subjective sleep and PM scores with markers of quality of life

|  | PSQI | ISI | EB | TB | Pro-spective | Retro-spective | Associative |
| --- | --- | --- | --- | --- | --- | --- | --- |
| Whole group of participants | | | | | | | |
| STAI-B | 0.28 * | 0.31 * | -0.16 | 0.08 | -0.14 | -0.15 | 0 |
| BDI | 0.27 | 0.35 ** | -0.06 | 0.1 | -0.06 | -0.02 | 0.05 |
| BC patients | | | | | | | |
| FACT-G | -0.65 *** | -0.57 *** | 0.17 | 0.08 | 0.13 | 0.23 | 0.13 |
| FACIT-F | -0.59 *** | -0.51 ** | 0.3 | 0.03 | 0.16 | 0.38 * | 0.18 |

PSQI: Pittsburgh Sleep Quality Index; ISI: Insomnia Severity Index; EB: event-based; TB: time-based; STAI-B: State Trait Anxiety Inventory – Trait; BDI: Beck Depression Inventory; FACIT-F: Functional Assessment of Chronic Illness Therapy – Fatigue; FACT-G: Functional Assessment of Cancer Therapy – General. * *p* < 0.05; ** *p* < 0.01; *** *p* < 0.001.
